# Effects of fermentable high fiber diet supplementation on gut derived and conventional nitrogenous product in patients on maintenance hemodialysis: a randomized controlled trial

**DOI:** 10.1186/s12986-019-0343-x

**Published:** 2019-03-12

**Authors:** Hamid Tayebi Khosroshahi, Behzad Abedi, Morteza Ghojazadeh, Azam Samadi, Abolghasem Jouyban

**Affiliations:** 1 Biotechnology Research Center, Tabriz University of Medical Sciences, Tabriz, Iran; 2 Department of Nanotechnology, Tabriz University of Medical Sciences, Tabriz, Iran; 3 Research Center for Evidence- Based Medicine, Tabriz University of Medical Sciences, Tabriz, Iran; 4 Drug Applied Research Center, Tabriz University of Medical Sciences, Tabriz, Iran; 5 Pharmaceutical Analysis Research Center and Faculty of Pharmacy, Tabriz University of Medical Sciences, Tabriz, Iran; 6 ISIbama research group, Tabriz, Iran

**Keywords:** Chronic kidney disease, Gut microbiota, P-cresol, Short chain fatty acid, Dietary

## Abstract

**Introduction:**

Gut derived toxins such as p-cresol, p-cresyl sulfate (pCS) and indoxyl sulfate (IS), which belong to protein-bound uremic toxins that promote development of fibrosis inflammatory state associated with chronic kidney disease. One possible way to suppress the production of IS and pCS is to increase dietary fiber intake. The aim of the present study was to assess whether increasing dietary fiber, as high amylose diet, can affect the level of conventional and protein bound nitrogenous products.

**Methods:**

Fifty patients with end stage renal disease (ESRD) on maintenance hemodialysis were randomly assigned to receive a diet containing resistant starch (HAM-RS2) or placebo over 8 weeks spanning February and September 2017 in the 29 Bahman hospital hemodialysis ward in Tabriz, Iran. Of these, 44 patients (23 from HAM-RS2 and 21 control) completed the study. Plasma levels of urea, creatinine, uric acid and other routine parameters were measured at the beginning and after 8 weeks of starting the supplementation. The levels of IS and p-cresol in the collected serum samples were also determined by HPLC at baseline and after intervention.

**Results:**

There was significant reduction of creatinine and uric acid levels in HAM-RS2 supplemented patients when compared with control group (*P* < 0.05). Serum levels of IS was not changed significantly in both HAM-RS2 treated and control patients, whereas p-cresol level was reduced significantly during the study period in HAM-RS2 treated patients (*P* = 0.039). The change of other parameters including Hb, lipids, bone markers and hs-CRP were non-significant during the study in both groups.

**Conclusion:**

Administration of fermentable high fiber diet as HAM-RS2 decreased serum levels of some nitrogenous products such as serum creatinine and p-cresol as a gut derived nitrogenous product without change in IS levels in maintenance hemodialysis patients. Due to safety, without important side effects the administration of diet enriched with fermentable fiber is suitable for patients on maintenance dialysis.

## Introduction

It has been established that beside high blood pressure and diabetes, also disruption of normal gut microbiota (dysbiosis) and derived inflammation, uremic toxicity and oxidative stress are playing a role in the pathogenesis of chronic kidney disease (CKD) and CKD-associated complications [1, 2]. Uremia and dietary changes (e.g. potassium-rich fruits free and low vegetables diet) are two implicit factors that cause dysbiosis of the gut microbial, which followed by production of several urease- possessing bacteria including ammonia, phenols, indoles and p-cresol [1, 3]. Bacterial flora derived toxins such as p-cresol, p-cresyl sulfate (pCS) and indoxyl sulfate (IS) belong to protein-bound uremic toxins that activate innate immunity and development of fibrosis, and hence promote the inflammatory state associated with CKD [4, 5]. Among the mentioned toxins, p-cresol is predominantly produced by bacterial flora of the distal colon (*e.g*, *Clostridium bartletti*, *Clostridium butyricum*, *Clostridium perfringens*, and *Fusobacterium* sp. etc.) during aromatic amino acid fermentation metabolism [6]. Since IS and pCS are protein bounded, they are poorly can be cleared by hemodialysis (HD) [7].Given that previous studies reported that p-cresol dysregulates hemostasis function of blood cells, endothelial cells. In addition it has been proposed that elevated p-cresol concentration results in increased overall mortality and cardiovascular diseases in nondiabetic hemodialysis patients [8, 9]. Decreased ingestion of dietary fiber can damage the mechanical wall of the gut, which triggers dysbiosis and bacterial translocation into the blood and micro-inflammation [10]. On the other hand, high fiber diet can increase the production of short chain fatty acids (SCFA), which is a crucial nutrient for regulatory T lymphocytes (T reg) activation [11]. As T reg regulates intestinal immune system homeostasis, hence any dysregulation of this pathway will associate with diet-related chronic inflammatory diseases, such as seen in CKD [7]. In addition to T reg, inflammation is augmented via migration of monocytes and macrophages to the sites of inflammation [12].

Furthermore, results from previous studies suggest that high dietary fiber therapy may alleviate the progression of renal injury and kidney dysfunction in patients with CKD. This hypotheses studied in animal model through studying the impact of dietary fiber, high amylose maize resistant starch type 2 (HAM-RS2), in a rodent model of CKD with promising results [3] but has not been reported in patients with CKD or on HD. One possible approach to suppress the production of IS and pCS is to increase the dietary fiber intake [13, 14].

The aim of present study is to further investigate the effects of diet enriched with resistant starch type 2(HAM-RS2) on serum levels of conventional and gut microbiome-derived nitrogenous waste products including p-cresol and IS in the patients on maintenance hemodialysis.

## Methods and materials

### Study design

In a double-blind controlled randomized clinical trial, the effects of ingestion of resistant starch type 2 (HAM-RS2) enriched diet was compared with placebo on conventional and gut derived nitrogenous waste products (p-cresol and IS) in End-Stage Renal Disease (ESRD) patients on maintenance hemodialysis. The study was conducted for 8 months from February to September 2017 in the 29 Bahman hospital hemodialysis ward in Tabriz, northwest of Iran. The study protocol was approved by the Human Subjects Institutional Review Board at Tabriz University of Medical Sciences (TUOMS), Tabriz, Iran (IR.tbzmed.rec.1397.442) and conducted in accordance with the Declaration of Helsinki. The trial has been registered at Iranian Registry of Clinical Trials website^1^ (IRCT 2016062628644 N1). Informed consent was obtained from all participants.

### Participants

Patients who were on maintenance hemodialysis thrice-weekly for at least 6 months and had at least 18 years of age were enrolled to the study. Patients who had diabetes, gastrointestinal diseases, active inflammatory disorders, infections, malignancies, changes in dialysis planning or pattern, or those who have received antibiotics prior to the enrollment were excluded from the study.

The sample size was calculated based on previous information from a pilot study recently conducted by our research group. During the pilot phase, 5 patients were recruited to each arm of study; 5 in intervention group (HAM-RS2) and 5 in placebo group. P-Cresol was set as the primary outcome measure and according to a between group mean difference of 1.5 and *α*=0.05 and ß = 0.2, considering a 10% falling out rate, final sample size was estimated as 25 patients each group (a total of 50 patients). Then 50 patients were enrolled in the study and were randomly allocated to two groups: Intervention (HAM-RS2) and comparison (placebo) using a randomization list generated by Randlist software (version 1.2), resulting 25 patients in each group. As a double-blind study, the patients and clinical investigators were blinded from knowing the per-patient treatment regimen.

High fiber diet and placebo were prepared as cracker by Araspar Benis Inc. (Tabriz, Iran). HAM-RS2 (60% pure) was purchased from Ingredion ANZ Pty Ltd., Lane Cove, NSW, and Australia. The crackers had the same rectangular shape, weight (60 g) and calories, which contained 20 g or 25 g of 60% resistant starch in HAM-RS2 enriched crackers and 20 g or 25 g of waxy corn starch as the placebo that ingested during hemodialysis and at home between main meals. The subjects’ energy requirements estimated from the basal metabolic rate in both groups. The treatment group took supplements for 8 weeks. In order to increasing the intestinal tolerability and reducing the probable side effects in treatment group, we administered crackers with 20 g resistant starch (RS) for the first 4 weeks and then 25 g for the second 4 weeks. The control patient received crackers with the same shape, weight and calorie, which prepared with waxy corn starch without using high amylose. The change of RS did not accompanied with change of the macronutrient composition and there was no dietary changes along with RS administration during the study.

Data on bowel habits and gastrointestinal symptoms were recorded using a checklist that included questions about the nausea, vomiting, abdominal pain, intestinal transit frequency/ week and blowing.

### Laboratory tests

Approximately, 10 mL of the peripheral blood samples were drawn out before beginning of the study and after 8 weeks at the end of study period. All samples were taken in the morning in the fasting state before the hemodialysis session. The blood samples were immediately centrifuged at 3000 rpm for 10 min and serum was separated and divided in two aliquots. The first set was processed for determination of urea nitrogen, creatinine, uric acid, Hb, Hct and bone markers. The second set was stored at − 80 °C and processed for measurement of p-cresol, IS and hs-CRP.

Serum concentrations of p-cresol and IS were measured using a high performance liquid chromatography with fluorescence detector (HPLC-FL). Briefly, serum samples were deproteinized by hydrochloric acid and heat denaturation. HPLC-FL analyses were performed using a LC apparatus (Knauer, Germany) and a 250 × 4.6 mm Nucleosil® C18 column of 5 μm particle size. The mobile phase, which consisted of sodium acetate buffer (pH 3.8) and acetonitrile (20:80, *v*/v), was delivered at flow rate of 1.3 mL min^− 1^. The excitation/emission wavelengths were set at 280/375 nm and 284/310 nm for IS and p-cresol, respectively. Standard curves for both compounds were set at 0.5, 1, 2.5, 5, 8 and 10 mg L^− 1^. The hs-CRP concentration was determined using an immune turbidimetric assay (BIOSYSTEMS, Spain).

### Statistical analysis

Normal distribution of data was verified with the Kolmogorov-Smirnov test. To normalize the data distribution, a logarithmic conversion was used for IS and serum creatinine level. Between-group comparisons of routine blood tests were carried out by repeated measures of ANOVA. In addition, analysis of covariance (ANCOVA) was used for comparing p-cresol and IS before and after intervention between the study groups. Intra-group comparison was performed using paired t-test and also Wilcoxon test for before-after comparisons in each group. The baseline levels were considered as covariate in Repeated Measures ANOVA and ANCOVA. Statistical analysis was performed using SPSS for Windows (SPSS, Chicago, IL, USA). Intention-to-treat and per-protocol analysis was performed based-on obtained data. All the tests were performed 2-tailed and *P* value < 0.05 was considered statistically significant.

## Results

### Clinical data

**Fig. 1:**
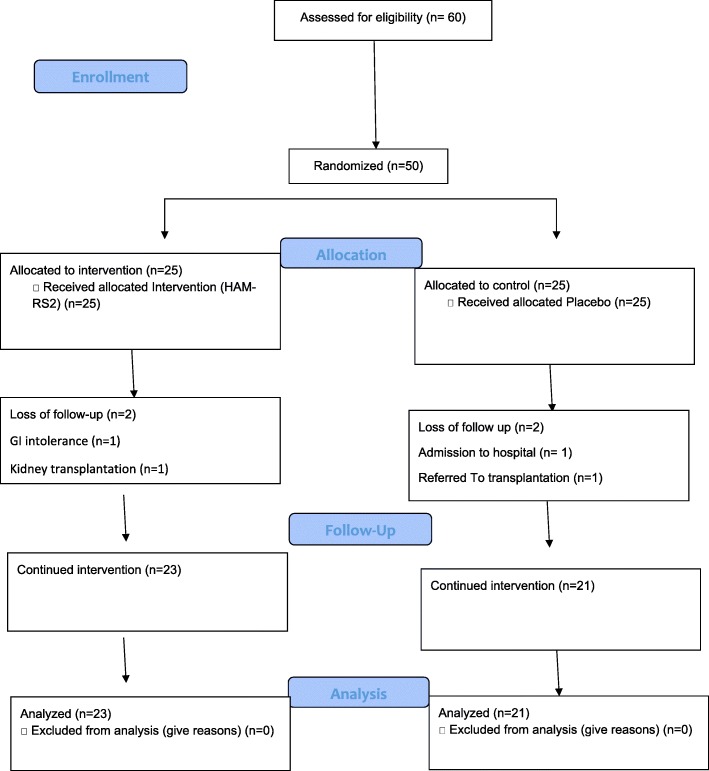
CONSORT 2010 Flow Diagram of trial

From a 50-patient set of entries, two patients from HAM-RS2 group (one patient because of gastrointestinal intolerance and one patient referred for kidney transplantation) and four patients from controls (two patients admitted to hospital, one patient missed dialysis session and one patient sent to other dialysis center) were dropped out of the study. Finally, 23 patients in HAM-RS2 group and 21 patients in control group reached the final step and were followed for end-point data collection. Figure 1 shows the enrollment, randomization, allocation and follow-up process of the current study as a flow diagram.

Mean age of patients was 53.17 ± 10.15 years for HAM-RS2 group and 57.90 ± 13.34 for controls. Gender distribution (M/F) was 14/11 and 15/10 in HAM-RS2 and placebo groups, respectively. Age, gender distribution, body-mass index (BMI) and hemodialysis duration had no significant difference between groups, Table 1.

**Table 1:** Demographic characteristics

	Intervention Group	Control Group	*P* value
	[HAM-RS2] (*n* = 25)	[Placebo] (n = 25)	
Age^a^_(years)_	53.17 ± 10.15	57.90 ± 13.34	0.182
Gender (Male/Female)	14/11	15/10	0.30
Body mass index^a^_(BMI)_	24.40 ± 2.2	23.86 ± 1.6	0.41
Hemodialysis duration^a^_(years)_	5.12 ± 1.25	4.78 ± 1.75	0.18

^a^Data are shown in mean ± SD

Laboratory data were collected and analyzed in within-group (before-after) and between group (before-before, after-after, Mean-difference) manners. According to Table 2, there was not any significant changes in serum levels of laboratory data shown in Table 2 before and after placebo administration in control group, but in intervention (HAM-SR2) group the reduction in serum creatinine (*P* < 0.006) and uric acid (*P* < 0.004) levels were statistically significant after the intervention. Serum urea concentration was also reduced in treated group, albeit none significantly, in this study (*P* = 0.09). Changes of other parameters of intervention and control groups were not significant. Results of between-group analyses are shown in Table 2.

**Table 2:** Comparison of laboratory data within groups and between groups

Laboratory Data	Intervention group (HAM-RS2)		Control group (placebo)		*p*-value	ES
	before	after	Before	after		
SUN	57.69 ± 13.63	52.34 ± 14.08	58.95 ± 16.13	58.61 ± 14.72	0.090	0.43
Creatinine	8.51 ± 2.05	7.48 ± 1.39	8.83 ± 2.45	9.35 ± 2.17	0.006	0.82
Uric acid	7.17 ± 1.11	6.59 ± 1.08	7.85 ± 1.00	7.16 ± 1.16	0.004	0.50
calcium	8.46 ± 05	8.66 ± 0.39	8.50 ± 0.64	8.38 ± 1.36	0.081	0.27
phosphorus	4.42 ± 0.94	4.63 ± 0.98	4.89 ± 1.12	5.28 ± 1.09	0.301	0.62
cholesterol	155.86 ± 37.93	159.12 ± 42.42	163.33 ± 36.85	146.17 ± 37.51	0.098	0.32
Triglyceride	143.91 ± 65.81	142.22 ± 87.06	154.40 ± 69.12	140.22 ± 73.48	0.707	0.02
albumin	4.46 ± 0.42	4.30 ± 042	4.54 ± 0.44	4.31 ± 0.33	0.901	0.02
HDL	44.17 ± 8.04	45.73 ± 10.90	46.19 ± 14.64	44.80 ± 13.19	0.449	0.07
iPTH^a^	293.00 (148.00455.0)	238.00 (152.00–448.00)	185 (151.00–496.00)	232.00 (148.50–456.00)	0.370	0.04
Hb	11.52 ± 1.01	11.86 ± 1.41	11.49 ± 1.150	12.99 ± 4.476	0.217	0.34

All Data are shown in mean ± SD

^a^Data are shown in median (Q_1_-Q_3_)

The underlying causes of ESRD were hypertensive nephrosclerosis (*n* = 28), glomerulonephritis in (*n* = 8), chronic interstitial nephropathy (*n* = 6), polycystic kidney disease (*n* = 1) and CKD of unknown etiology (*n* = 3). All of the patients were on the same medications including erythropoietin, iron, vitamins, antihypertensive, and phosphate binders. No significant difference was present in other demographic data includes dialysis vintage, causes of renal failure, dialysis access. Inter-dialysis weight gains were 2.65 kg versus 2.45 kg in the HAM-RS2 group and 2.42 kg versus 1.99 kg in placebo group, before and at the end of study, respectively (*P* > 0.05). Likewise, no significant difference was observed in the mean weight and BMI before (24.40 ± 2.2 v.s. 22.92+/− 1.8) and at the end (23.86 ± 1.6 vs. 21.41+/− 2.1) of the study in HAM-RS2 group and placebo-treated group, respectively. The frequency of intestinal transit were similar during the 4 weeks prior to the onset of study in the HAM-RS2 and control groups (3.5 v.s. 3.6 per week, respectively), but increased in the HAM-RS2 compared with the control group (4.2 v.s. 3.7 per week, respectively) at the end of study. Data derived from the Medical Outcomes Study Questionnaire including Kidney Disease and Quality of Life (KDQOL™-360) showed no significant change during the study in either HAM-RS2 or control group. There was mild to moderate side effects including abdominal discomfort (distention *N* = 2 and nausea N = 2) in four patient from HAM-RS2 group and two patients (dyspepsia and nausea) from control. Only one patient in HAM-RS2 group had experienced severe adverse effects during the intervention, including epigastric pain and vomiting after 3 weeks of drug administration, therefore the intervention discontinued and the patient was excluded from study.

### General laboratory data

Routine laboratory Data are shown in Table 3. HAM-RS2 group exhibited a significant time-dependent reduction in serum urea nitrogen and creatinine and uric acid concentrations (*P* < 0.05). However, no significant changes were observed in serum urea, creatinine and uric acid concentrations in placebo group during the study period. No significant changes were found in other laboratory values including Hb, Hct, ferritin, TIBC, intact PTH, total cholesterol, HDL cholesterol or triglyceride in both groups. In addition, within-group analysis of p-cresol serum level showed significant decrease in intervention group (*p* = 0.039) while there was increasing of p-cresol in control group that was non-significant (*P* = 0.86).

**Table 3:** Comparison of Laboratory Data within groups and between groups

Laboratory Data	Intervention Group (HAM-RS2)^*^		Control Group(Placebo)^*^		*P*-value^**^	ES
	Before	After	Before	After		
Indoxyl sulfate	37.42 (24.32–49.57)	38.53 (28.64–50.07)	40.75 (24.46–48.62)	45.26 (30.15–52.2)	0.606	0.14
p-Cresol	7.8 (2.29–15.67)	5.34 (3.01–11.57)	6.97 (1.48–14.62)	7.02 (2.52–18.9)	0.992	0.03
HS-CRP	4.7 (3.23–10.13)	9.9 (2.8–14.3)	4.87 (2.97–16.56)	6.09 (2.35–16.72)	0.866	0.04
Total Antioxidant	2.38 ± 0.43	2.24 ± 0.38	2.54 ± 0.29	2.24 ± 0.40	0.917	–

* Median(Q_1_-Q_3_) ** P-value of ANCOVA for between group comparison

hs-CRP concentrations were also not changed in the study population including the HAM-RS2 and control groups during the observation period. In addition, serum levels of IS were not changed significantly neither in HAM-RS2 group nor in controls (Table 3). Further, the same happened for hs-CRP levels.

## Discussion

Apparently, conventional dialysis modalities deliver inadequate clearance of organic solutes that accumulate in patients with renal failure, which probably contribute to poor health outcomes in chronic dialysis patients. Based on physiology, there are two ways to remove nitrogenous products: (a) urine and (b) feces. By reducing the protein amount and increasing availability to fermentable carbohydrate, nitrogenous elimination by fecal route excretion can be increased [13, 15]. Significant number of uremic solutes is derivate from function of the intestinal microbium. IS and p-cresol are the two most largely studied solutes that derive from intestinal microbes’ activity. Much evidence suggest that they are toxic [16–20] and due to significant protein binding, they are poorly dialyzable by conventional dialysis [7, 21]. One of the possible ways to reduce the production of solutes derive from colon microbial breakdown of amino acids is to increase dietary fiber [13, 21]. Yet little is known about the effects of fermentable carbohydrate on protein bounded and gut produced nitrogenous products. Recent studies indicated that intestinal microbium were changed in dialysis patients [22, 23]. We did not investigate the composition of the intestinal microbiome in this study. However, recently a new study regarding the intestinal microbiota of maintenance hemodialysis patients and effect of dietary fiber is begging by our research team.

During the last decades, dietary fiber has been used as supporter to restriction of dietary protein in patients with renal failure [24–26]. Our study showed significant reduction of the serum concentration of p-cresol with consumption of diet enriched with HAM-RS2 in patients on maintenance hemodialysis. However, IS level didn’t change significantly in the treatment group. There are some recent studies about the effects of high fiber diets on gut derived waste products with conflicting results [27–32].

In a comparative study, consuming of resistant starch reduced free IS levels at a dose, which did not increase gastrointestinal symptoms above the level seen with a control starch in hemodialysis patients [33]. Since administration of HAM-RS2 diet can induce the luminal pH, it is estimated that increased production of SCFA, decrease production of IS and p-cresol sulfate [3]. It is proposed that complex carbohydrates in resistant starch can work as the substrates for production of SCFA [34, 35] by symbiotic microbes [29] that whose population is significantly reduced in patients with chronic renal failure. In another study by Meijers et al. there was 20% reduction of plasma level of p-cresol sulfate but had no effect on IS by the administration of oligofructose-enriched inulin. They administered a dose of 10 g enriched inulin daily for the first week followed by 10 g twice daily for the next 3 weeks. The results of this study was consistent with our results regarding the independent change of P cresol and IS after high fiber diet administration. These finding showed that although IS is also generated by colonic bacteria, serum concentrations was not decreased with high fiber diet. This may be due to the fact that P cresol and IS are end products of unrelated bacterial metabolic pathway. This explains that the serum concentration of P cresol and IS changed independently [13].

Based on physiology, there are two ways to remove nitrogenous products: (a) urine and (b) feces. By reducing the protein amount and increasing availability to fermentable carbohydrate, nitrogenous elimination by fecal route excretion can be increased [13, 15]. In addition, the administration of oligosaccharides induced a 20 to 30% decrease in blood urea and renal nitrogen excretion relative to the control, indicating a potential for oligosaccharide diet therapy in patients with chronic renal disease [26, 36].

Conventional nitrogenous products have been reduced significantly in our dialysis populations with consumption of fermentable complex carbohydrate. Based on the results of the present study, serum level of creatinine and uric acid were reduced significantly in HAM-RS2 treated patients *p* < 0.05. In addition, serum urea nitrogen (SUN) was reduced from 58.32 ± 13.62 to 52.27 ± 14.41 mg/dL during 2 months of HAM-RS2 intake when compared with control patients [*P* = 0.09]. The causes of reduction of conventional waste products by high fiber diet is not yet completely resolved. Previous studies suggested that increasing fiber intake can reduce the plasma concentrations of urea in patients with renal failure by causing a larger portion of ingested nitrogen to be excreted as microbial proteins in the stool [12, 26–28]. In a study by Rampton, et al. 6 and 8 week courses of two different hemicelluloses, arabinogalactan and ispaghula, decreased mean plasma urea in uremic patients by 11 and 19% respectively. The decrease in plasma urea caused by dietary fiber is probably to be due to inhibition of colonic bacterial production of ammonia; and they suggested that, such therapy could alleviate some of the symptoms of uremia [24]. A number of observational and in vitro studies had evaluated the effect of dietary fibers (prebiotics) on uric acid. The Results of these studied show that prebiotics lower uric acid levels through mechanisms that are largely unknown. In addition, some data suggested that dietary fibers can reduced serum levels of uric acid and urea nitrogen concentrations in serum by attenuating the absorption of dietary adenine. [37, 38]

Beside the change in the fiber content of diet, other manipulations can also reduce nitrogenous products level by reducing the production of gut derived protein bound products or increased intestinal elimination of conventional nitrogenous products. In a recent study by our team, significant reduction in serum level of nitrogenous products were observed with ingesting of 8 week lactulose as a prebiotic [39]. In addition, consumption of probiotics may change the microbiome and reduce the production of undesirable compounds. Others methods to reduction of protein bound nitrogenous products are use of charcoal sorbent AST-120 and α-glucosidase inhibitor acarbose that reduces the levels of IS and p-cresol [40, 41].

In conclusion, this study showed that diet enriched with fermentable high fiber as HAM-RS2 can decreased serum levels of some nitrogenous products like serum creatinine and uric acid. Gut derived nitrogenous products like p-cresol was also reduced in HAM-RS2 treated patients without change in IS in maintenance hemodialysis patients. In addition, there was no important side effects including gastrointestinal intolerance with prescribed doses in our HAM-RS2 treated patients when compared with control ones. Based on the results of this study and other recent studies and safety of diet enriched with fermentable fiber, and known toxic effect of gut derived nitrogenous products especially cardiovascular toxicity, the administration of this diet is advisable in maintenance hemodialysis patients.

### Study limitations

This study had some limitations. We did not measure the intestinal microbiota changes in this study but we conducting other study for evaluation of the effect of high fermentable diet on intestinal microbium separately. The patient population and duration of study were limited in this study. We did not studied diabetic patients in this study that may had more benefit from this treatment.

## Footnotes

1
www.irct.ir
